# Phosphorus May Induce Phenotypic Transdifferentiation of Vascular Smooth Muscle Cells through the Reduction of microRNA-145

**DOI:** 10.3390/nu15132918

**Published:** 2023-06-27

**Authors:** Sara Fernández-Villabrille, Beatriz Martín-Carro, Julia Martín-Vírgala, Cristina Alonso-Montes, Alejandra Fernández-Fernández, Carlos Martínez-Salgado, José L. Fernández-Martín, Manuel Naves-Díaz, Jorge B. Cannata-Andía, Natalia Carrillo-López, Sara Panizo

**Affiliations:** 1 Metabolismo Óseo, Vascular y Enfermedades Inflamatorias Crónicas, Instituto de Investigación Sanitaria del Principado de Asturias (ISPA), 33011 Oviedo, Spain; 2 Redes de Investigación Cooperativa Orientadas a Resultados en Salud (RICORS2040, Kidney Disease), 28040 Madrid, Spain; 3 Laboratory of Medicine, Hospital Universitario Central de Asturias, 33011 Oviedo, Spain; 4 Institute of Biomedical Research of Salamanca (IBSAL), 37007 Salamanca, Spain; 5 Bone and Mineral Research Unit, Hospital Universitario Central de Asturias, 33011 Oviedo, Spain; 6 Department of Medicine, Universidad de Oviedo, 33011 Oviedo, Spain

**Keywords:** phosphate, vascular calcification, miR-145, serum biomarkers, osteogenic differentiation, α-actin

## Abstract

Phosphorus is a vital element for life found in most foods as a natural component, but it is also one of the most used preservatives added during food processing. High serum phosphorus contributes to develop vascular calcification in chronic kidney disease; however, it is not clear its effect in a population without kidney damage. The objective of this in vivo and in vitro study was to investigate the effect of high phosphorus exposure on the aortic and serum levels of miR-145 and its effect on vascular smooth muscle cell (VSMCs) changes towards less contractile phenotypes. The study was performed in aortas and serum from rats fed standard and high-phosphorus diets, and in VSMCs exposed to different concentrations of phosphorus. In addition, miR-145 silencing and overexpression experiments were carried out. In vivo results showed that in rats with normal renal function fed a high P diet, a significant increase in serum phosphorus was observed which was associated to a significant decrease in the aortic α-actin expression which paralleled the decrease in aortic and serum miR-145 levels, with no changes in the osteogenic markers. In vitro results using VSMCs corroborated the in vivo findings. High phosphorus first reduced miR-145, and afterwards α-actin expression. The miR-145 overexpression significantly increased α-actin expression and partially prevented the increase in calcium content. These results suggest that miR-145 could be an early biomarker of vascular calcification, which could give information about the initiation of the transdifferentiation process in VSMCs.

## 1. Introduction

Phosphorus (P) is a key element for life that is present in all cells of the organism. P is also found in most foods as a natural component, and it is one of the most used preservatives added during food processing. Progressively, over the last decades, an exponential increase in the consumption of processed foods has been observed, and in many circumstances the intake of P exceeds the dietary recommendations [1]. The seminal knowledge of the potential risks related to high P intake is based on studies in patients with chronic kidney disease (CKD) who display elevated serum P levels in the late stages of CKD, resulting in a potent stimulus that triggers secondary hyperparathyroidism [2], increases vascular calcification, aggravates left ventricular fibrotic hypertrophy leading to heart failure, accelerates kidney damage and increases the risk of mortality [3,4,5,6,7,8].

In individuals with normal renal function, results are less conclusive, although a positive association between the increase in serum P and the prevalence of cardiovascular disease has been found [9]. Furthermore, despite its importance, it is still unclear whether a high P exposure in the presence of normal renal function could influence the transdifferentiation of vascular smooth muscle cells (VSMCs) to bone-like cells, able to promote vascular calcification.

The VSMCs, mainly located in the medial layer of the arterial wall, are responsible for the contractile properties of the arteries [10,11]. Under several stimuli, VSMCs can transdifferentiate and lose the phenotypic markers responsible for smooth muscle cell contractility, such as α-actin—the main marker of the vascular and contractile phenotype of VSMCs—and differentiate to osteoblast-like cells which express proteins characteristic of bone [12], leading to vascular calcification, which is currently diagnosed once the crystals with calcium (Ca) and P have been already deposited. Thus, strategies must be designed to identify non-invasive and sensitive biomarkers of vascular calcification, such as microRNAs (miRs) that may allow to advance the diagnosis of vascular calcification at earlier stages.

miRs are small non-coding RNA molecules that play a crucial role in gene regulation and post-transcriptional control of gene expression [13]; they are good candidates as biomarkers, as they can be easily measured in serum, plasma or urine using simple and cheap techniques. Several studies have identified miRs as being important regulators of genes related to the cardiovascular complications [14,15,16]. In fact, low levels of miR-145—the most prolific miR in VSMCs—has been associated with loss of contractile phenotypes, increased osteogenic differentiation [17,18,19,20] and consequently, vascular calcification [21].

Therefore, the objective of the present study was to investigate the influence of high P exposure on the aortic expression and serum levels of miR-145 and its effect on the VSMC changes towards less contractile phenotypes.

## 2. Materials and Methods

The present work was carried out combining an in vivo and an in vitro approach; (a) an in vivo rat study, using aortas and serum from rats with normal renal function fed a normal and high-P diet and; (b) an in vitro study, conducted in VSMCs (A7r5) exposed to different concentrations of P and in VSMCs, in which miR-145 was overexpressed and silenced to elucidate its potential role in the VSMCs phenotypic transdifferentiation. 

The in vivo experimental protocol was approved by the Laboratory Animal Ethics Committee of Oviedo University (PROAE 14/2021) and it adhered to the National Institutes of Health Guide for the Care and Use of Laboratory Animals.

### 2.1. In Vivo Study

#### 2.1.1. Animal Model

Four-month-old male Wistar rats (350–400 g) were divided into two groups: one group fed a commercial rodent chow with a standard P content (NP; 0.6% P, 0.6% Ca and 20% protein content; Envigo, IN, USA) (*n* = 10), and a group fed a high P diet (HP; 0.9% P, 0.6% Ca, and 20% protein content) (*n* = 10). Rats were housed in wire cages and received diet and water ad libitum. After 18 weeks, rats were sacrificed by exsanguination using isoflurane anaesthesia, and serum samples were obtained for analyses. Aortas were removed, washed twice with saline solution, and cut into three pieces used for RNA/miR extraction, Ca content and paraffin inclusion. Hearts were also collected, washed three times with saline solution and weighted.

#### 2.1.2. Biochemical Markers

Serum and urinary Ca, P and creatinine were evaluated using automated chemistry analyzer (Hitachi 717, Boehringer Mannheim, Germany). Serum parathormone (PTH) and fibroblast growth factor-23 (FGF23) were measured by sandwich ELISA (Rat Intact PTH ELISA Kit; Quidel, San Diego, CA, USA and Mouse/Rat Intact FGF23 kit; Immutopics, San Clemente, CA, USA) conforming the manufacturer’s instructions. The fractional excretion of P and Ca was calculated as [(urine molecule × serum creatinine)/(serum molecule × urine creatinine)].

#### 2.1.3. Blood Pressure Measurement

On the week previous to the sacrifice, systolic (SBP) and diastolic blood pressure (DBP) were measured by non-invasive blood pressure monitoring in conscious restrained rats (tail-cuff technique (LSI Letica, Panlab, Barcelona, Spain). To reduce the stress induced by this procedure, animals were accustomed to the instrument for 4 consecutive days prior to the definitive measurements, consisting in a set of a minimum of 10 repetitive determinations per rat. Only stable, reproducible values were taken into account.

### 2.2. In Vitro Study

#### 2.2.1. Induction of A7r5 Osteogenic Phenotype Differentiation and Calcification

The cell line A7r5 (VSMCs from rat aorta, ATCC) was grown in Dulbecco modified eagle medium (1.8 mM Ca and 1 mM P) (DMEM, Lonza, Alsace, France) supplemented with 10% fetal bovine serum (FBS, Lonza, Alsace, France) and 1% penicillin/streptomycin (Lonza, Alsace, France) to subconfluence. Three different types of experiments were conducted:

(1) To evaluate the influence of the P concentration, A7r5 cells were cultured in DMEM with 1% FBS increasing the P content as follows: 1 mM P (control with no P supplementation, named from now onwards as non-calcifying medium), 1.5 mM P, 2 mM P, 2.5 mM P, 3 mM P (named from now onwards as calcifying medium) and 3.5 mM P, the culture medium was replaced daily. Ca deposition and gene/miR expression were assessed 3 days after the exposure to P.
(2) In order to study the sequence of the changes, A7r5 cells were cultured in DMEM supplemented with 1% FBS and exposed to 1 mM P (non-calcifying medium) and 3 mM P (calcifying medium), for 0, 6, 12, 24, 36, 48, 60 and 72 h. Ca deposition and gene/miR expression in A7r5 cells and miR-145 levels in culture medium were assessed at every time point of the experiment.
(3) In order to elucidate whether PTH, which is frequently associated to high P, has a direct effect on α-actin expression, miR-145 levels and Ca content in VSMCs, A7r5 cells were cultured in DMEM (supplemented with 1% FBS) and exposed to 1 mM P or 3 mM P adding in both cases PTH 1–34 at different concentrations, (from 10^−9^ M to 10^−7^ M, Sigma-Aldrich, St. Louis, MO, USA), replacing the culture medium daily. Ca deposition and gene/miR expression were assessed 3 days after the exposure to P and PTH.

All the experiments were replicated at least three times with three wells per condition.

#### 2.2.2. Transfection with Mimics or Antagomirs 

A7r5 cells were seeded at a concentration of 1 × 10^5^ cells per well in six-well plates (Corning Costar, NY, USA). At 60–70% confluence, cells were transfected overnight with 500 pmol of miR-145 mimic (for miR-145 overexpression), an antisense oligonucleotide of miR-145 (antagomir, to inhibit miR-145 expression), (Thermo Fisher Scientific, Waltham, MA, USA) using the DharmaFECT transfection reagent (GE Healthcare Dharmacon, Lafayette, CO, USA) according to the manufacturer’s guidelines. Transfection with a scrambled sequence (Thermo Fisher Scientific, Waltham, MA, USA) acted as a negative control, with the same condition as mimics and antagomirs. Cells were then exposed for three days to non-calcifying and calcifying medium. Afterwards, Ca content, miR-145 and α-actin gene expression were measured. All experiments were repeated three times.

### 2.3. Analytical and Technical Procedures 

#### 2.3.1. Quantification of Ca Content 

A fragment of the rat abdominal aorta was washed with phosphate-buffered saline (PBS) and homogenized in 0.6 N HCl with an Ultraturrax (OmniHT). A7r5 cells were also washed three times with PBS and 0.6 N HCl was added to the wells. Both tissues and cells were shaken at 4 °C for 24 h. Upon centrifugation, Ca content was measured in the supernatants by the ortho-cresolphtalein complexone technique (Sigma-Aldrich, St. Louis, MO, USA). Pellets were resuspended in lysis buffer (125 mM Tris, 2% SDS, pH 6.8) for protein extraction and quantification by DC protein assay (Bio-Rad, Hercules, CA, USA). Ca content was normalized to total cell protein and expressed as micrograms of Ca per milligram of protein.

#### 2.3.2. Von Kossa Staining

Von Kossa staining was performed in five µm aorta sections following standard protocols. After capturing the images with a digital microscope (DMRXA2, Leica Microsystems, Wetzlar, Germany) coupled with a Leica DFC7000 T camera (Leica Microsystems), a semiautomatic image analysis software (ImageJ 1.52p) was used to evaluate the aortic calcification.

#### 2.3.3. RNA and MicroRNA Isolation, Retrotranscription and Quantitative PCR

A piece of the rat abdominal aorta was homogenized with Ultraturrax (OmniHT) in TRI Reagent (Sigma-Aldrich, St. Louis, MO, USA) according to the manufacturer protocols. Total RNA from serum, culture medium and A7r5 cells was also extracted with TRI Reagent. RNA concentration was quantified by UV-Vis Spectrophotometry (NanoDrop Technologies, Wilmington, DE, USA). Retrotranscription was carried out with a High-Capacity cDNA Reverse Transcription Kit (Applied Biosystems, Waltham, MA, USA) for small RNAs and genes. 

Real time quantitative PCR (RT-qPCR) was carried out using TaqMan Assays (Applied Biosystems, Waltham, MA, USA) in Quant Studio 3 (Applied biosystems, Waltham, MA, USA) to measure miR-145 and endogenous rat small nuclear RNA U6 or the spike in cel-miR-39 (Thermo Fisher Scientific, Waltham, MA, USA) that were used as references for tissue/cells and for serum/medium samples, respectively. α-actin, and osteogenic Runt-related transcription factor 2 (Runx2) and Osterix (Osx) gene expression were also determined by qRT-PCR and glyceraldehide-3-phosphate-dehydrogenase (GAPDH) was used as the housekeeping gene. 

miRs and target genes gene expression were evaluated by comparing threshold cycles using the ΔΔ cycle threshold method [22].

#### 2.3.4. Statistical Analyses

Data are shown as median and interquartile range (IQR). Groups were compared using the parametric Student’s t-test or the non-parametric Mann–Whitney–Wilcoxon U test for variables that followed normal or non-normal distribution. For multiple groups, the comparisons were performed using the analysis of variance (ANOVA) or Kruskal–Wallis with the post-hoc analysis Tukey’s or Dunn’s Multiple Comparison tests for normal or non-normal distributions, respectively. Pearson’s correlation was used to analyze the linear relationship of the data. All analyses were performed using R software 4.1.1.

## 3. Results

### 3.1. In Vivo Study

#### 3.1.1. Biochemical Markers

The rats fed a high-P diet (HP) showed significant higher levels of serum P and PTH than rats fed a normal-P diet (NP). No significant differences were found in serum levels of Ca, FGF23 and creatinine clearance (Table 1). The fractional excretion of P was remarkably higher in the rats fed a high-P (HP) diet and the fractional excretion of Ca was lower than in rats fed a normal-P diet (NP) (Table 1).

#### 3.1.2. Cardiovascular Parameters

DBP was significantly higher in the group of HP rats; however, no differences in SBP and heart/body weight ratio were found. There were no differences in the Ca content of the aorta and all samples were negative for Von Kossa staining. A significant decrease (51%) in the aortic α-actin expression was observed in the aortas from the HP group (Table 2). No changes in the osteogenic markers Runx2 and Osx were found.

**Figure 1:**
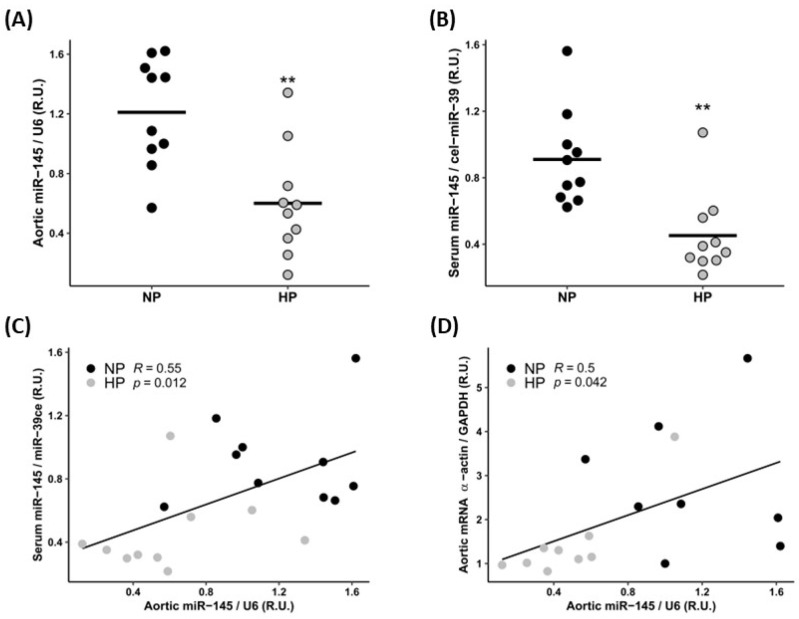
Aortic and serum miR-145 levels in rats fed a normal-P (NP) and high-P (HP) diet. Aortic (**A**) and serum (**B**) miR-145 levels. Correlation between aortic and serum miR-145 levels (**C**) and between aortic miR-145 levels and aortic α-actin gene expression (**D**). R.U. Relative units. Horizontal lines show the median. Black and grey spots represent rats fed normal-P diet (NP) and high-P diet (HP), respectively. ** *p* < 0.01 vs. NP group.

Aortic and serum miR-145 levels were significantly lower in HP rats (Figure 1A,B), and a significant positive correlation was found between both parameters (R = 0.55, *p* = 0.012; Figure 1C). The aortic α-actin expression showed a positive and significant correlation with aortic miR-145 levels (Figure 1D).

### 3.2. In Vitro Study

#### 3.2.1. Effect of Increasing the P and PTH Concentrations on miR-145 Levels, Phenotypic Marker Expression and Calcification of the VSMCs

**Figure 2:**
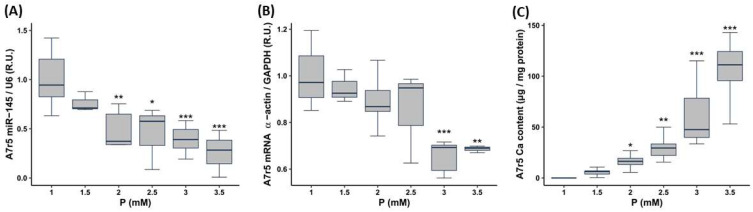
Effect of different P concentrations (from 1 to 3.5 mM) on A7r5 cells cultured for 72 h. miR-145 levels (**A**), α-actin gene expression (**B**) and Ca content (**C**). R.U. Relative units. Data represent median [interquartile range]. * *p* < 0.05, ** *p* < 0.01, *** *p* < 0.001 vs. 1 mM P.

A7r5 exposure to progressive increments in P concentration (from 1 to 3.5 mM P) caused a P concentration-dependent decrease in the miR-145 levels (statistically significant from 2 mM P upwards, Figure 2A) and in α-actin gene expression (statistically significant from 3 mM P upwards, Figure 2B). The Ca content increase was statistically significant from 2 mM P upwards (Figure 2C). The expression of the osteogenic markers Runx2 and Osx significantly increased from 3 mM P upwards (Supplementary Figure S1A,B)

The supplementation with PTH had no effect on either miR-145 levels or α-actin expression (Supplementary Figure S2A,B). Only 3 mM P + 10^−7^ M PTH induced a significant increase in Ca content compared to 3 mM P + 0 PTH (Supplementary Figure S2C).

#### 3.2.2. Time Course of miR-145 Levels, α-Actin Expression, and Calcification of the VSMCs Exposed to Standard and High P Concentration

**Figure 3:**
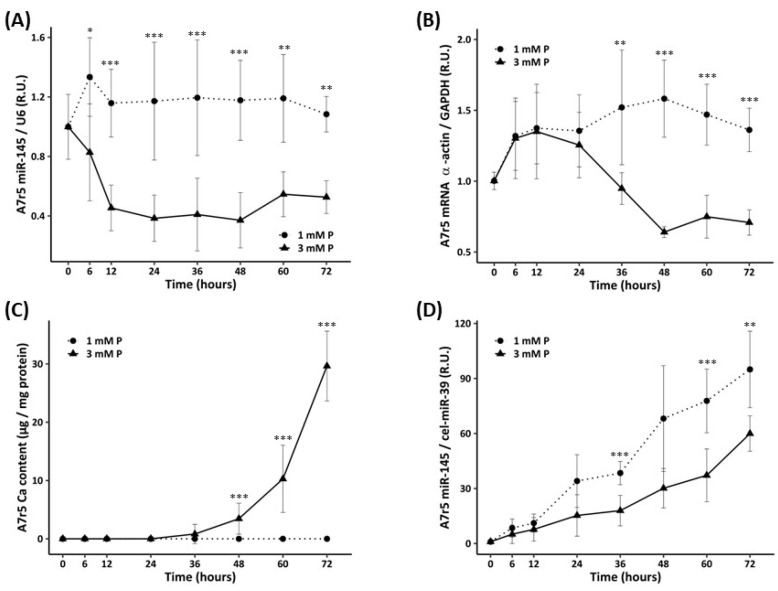
Time course (up to 72 h) of A7r5 cells exposed to a high concentration of P (3 mM). Intracellular miR-145 levels (**A**), α-actin gene expression (**B**), Ca content (**C**), miR-145 levels released into the culture medium (**D**). R.U. Relative units. Data represent mean ± standard deviation. Circles and dotted line represent the 1 mM P condition, whereas triangles and solid line represent the 3 mM P condition. * *p* < 0.05, ** *p* < 0.01, *** *p* < 0.001 vs. its own control 1 mM P.

Exposure of A7r5 cells to calcifying medium (3 mM P) resulted in a significant decrease in miR-145 expression (after 6 h of exposure), and in α-actin gene expression (after 36 h of exposure), compared both with non-calcifying medium conditions (Figure 3A,B). A7r5 cultured with calcifying medium showed a significant increase in Ca (after 48 h of exposure), compared to the non-calcifying medium (Figure 3C). miR-145 levels increased in a time-dependent manner in non-calcifying and calcifying medium, but after 36 h the miR-145 increase in the calcifying medium was significantly lower than in the non-calcifying medium (Figure 3D).

#### 3.2.3. Effect of the miR-145 Silencing and Overexpression on the Phenotypic and Calcification Changes of VSMCs 

The exposure of A7r5 cells to miR-145 antagomir reduced the miR-145 expression (Control 1.00 ± 0.23 R.U.; miR-145 antagomir 0.09 ± 0.03 R.U.), and the exposure to miR-145 mimic increased the miR-145 expression (Control 1.00 ± 0.23 R.U.; miR-145 mimic 84.00 ± 24.88 R.U.). 

**Figure 4:**
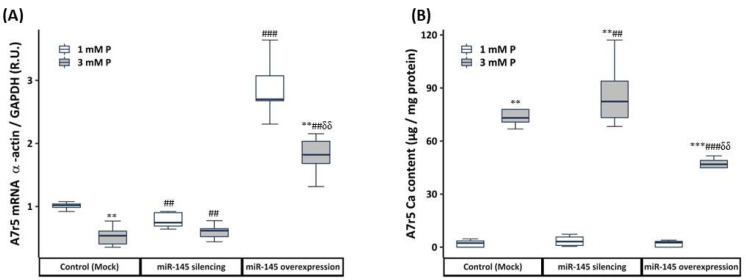
Effect of miR-145 silencing and overexpression on the phenotypic changes and calcification of A7r5 cells exposed to non-calcifying (1 mM P) and calcifying (3 mM P) medium. α-actin gene expression (**A**) and Ca content (**B**). R.U. Relative units. Data represent median [interquartile range]. White and grey boxes represent non-calcifying and calcifying medium, respectively. ** *p* < 0.01, *** *p* < 0.001 vs. its own control 1 mM P. ## *p* < 0.01, ### *p* < 0.001 vs. Control (Mock) 1 mM P. δδ *p* < 0.01, vs. Control (Mock) 3 mM P.

miR-145 silencing in non-calcifying medium induced a 23% reduction in α-actin expression without significant changes in Ca content (Figure 4A,B), and the miR-145 overexpression caused a 2.70-fold increase in α-actin expression without changes in the Ca content (Figure 4A,B). miR-145 silencing in calcifying medium did not change the α-actin expression and Ca content (compared to Mock 3 mM P, Figure 4A,B). miR-145 overexpression in calcifying medium, induced a 1.81-fold increase in α-actin expression, and partially prevented the increase in Ca content (compared to Mock 3 mM P, Figure 4A,B).

## 4. Discussion

The in vivo results of the present study showed that in rats with normal renal function fed a high P diet, a significant increase in serum P was observed despite a 103-fold increase in the fractional excretion of P, which nicely correlated with the significant serum PTH increase, but not with the minor and not significant FGF23 serum changes. These findings were associated to a significant decrease in the aortic α-actin expression, which paralleled the decrease in aortic and serum miR-145 levels, with no changes in the osteogenic markers. The in vitro results in VSMCs corroborated the in vivo findings. The increase in P reduced, in a concentration-dependent manner, miR-145 and α-actin expression. Furthermore, miR-145 overexpression significantly increased α-actin and partially prevented the increase in Ca content. 

The intake of P has increased worldwide, but unfortunately a great part of this increment is mainly due to the increased consumption of processed foods [1,23]. The inorganic P is a cheap good food preservative; thus, it is one of the main components used in the production of several processed foods such as, carbonated soft drinks, breakfast cereals, cookies, pastries, processed cheeses, instant products, frozen meals, sausages and several types of foods [24,25,26]. Another recently identified source of hidden P is the excipients used in the preparation of pharmacological products [27]. The toxic effects of P in several organs were first observed in patients with CKD, in whom the kidney removal of P is insufficient, and as a result, P accumulates. However, more recently, studies in general populations without renal failure have drawn attention to the fact that minor P serum increments, even in the upper range of normal serum P, could be harmful for the cardiovascular system and it has been associated with a higher risk of mortality [28,29,30]. 

The in vivo results of the present study support the clinical-epidemiological findings observed in individuals without renal impairment. In fact, the HP group, fed with a 50% P excess diet (0.9 vs. 0.6% of P), showed a significant increase in serum P despite the remarkable kidney efforts to remove P, with an 100-fold increase in the fractional excretion of P, which was driven by the significant increase in PTH but not by FGF23. The observed dissociation between PTH and FGF23 levels suggests that with normal renal function, PTH is the main regulator of P excretion, and FGF23 may play a secondary role, in contrast with what would happen in the CKD setting. In normal conditions, serum P is maintained in a narrow range through the combined regulatory actions of PTH, FGF23 and 1,25 dihydroxyvitamin D_3_. In CKD, the progressive reduction of glomerular filtration rate triggers the main compensatory mechanisms that involves an early and highly significant increase in serum FGF23, before the increments in serum PTH are observed. The sequence and magnitude of changes in FGF23 and PTH observed in CKD seem to be different to what happens with normal renal function. The comparison between the kidney handling of P in CKD and in the presence of normal renal function is still a matter of debate; unfortunately, studies in healthy individuals or normal rats and mice are scarce.

A similar situation occurs with the link between dietary P intake and blood pressure, in which the results are still controversial. In our rat model, high P intake induced a slight increase in arterial blood pressure, but only the increase in DBP was statistically significant. By contrast, a similar study, using a rat model with normal renal function and high P intake, showed an increase in arterial blood pressure which was explained through the increments of renin mediated by the rise in PTH [31]. Similarly, in the Premiere study, a human randomized behavioral intervention study, a high P intake was associated with increased SBP and DBP [32]. In an attempt to explain these differences, a secondary analysis of the Premiere study suggested that the controversial results of the effect of P on blood pressure could be partly explained by the different sources of P (organic P—plant and animal foods, or inorganic P), which might exert differential effects in the association between P intake and blood pressure [32]. In fact, the absorption of P from food may vary according to its origin: ranging between 10–30% in the vegetables, 40–60% in the animal products and 90–100% in the processed foods [24].

High serum P has been also associated with increased vascular calcification, and most of this knowledge came from studies carried out in CKD patients or in experimental CKD [4,33,34]. A systematic metanalysis including 10 cross-sectional studies assessing the relation between serum P and vascular calcification in adult healthy population found significant associations in eight of them [9]. Previous results from our group in rats with a severe kidney insufficiency (7/8 nephrectomy) have shown that 18 weeks, and even less time, were sufficient to induce vascular calcification, in rats fed a high-P diet [21,35,36].However, in the present study rats with normal renal function fed a standard or high-P diet for 18 weeks did not show significant differences in vascular calcification, probably due to the fact that rats with normal renal function need a longer P exposure to obtain Ca and P accumulation in the aorta. However, it is relevant to stress that the calcification process occurs in several steps ending with Ca and P deposited in the arteries. 

This step-by-step, pathophysiological process first requires the loss of the contractile phenotypes of the VSMCs; afterwards, the VSMCs change to osteogenic phenotypes, and finally the deposition of Ca and P. Thus, in the very early stages of this process, the first change expected is the loss of the VSMCs’ contractile properties which can be quantified by measuring α-actin, which represents 40% of the total cellular protein of VSMCs and conferres the contractile properties of the VSMCs [37]. In the present study carried out in rats with normal renal function, a significant reduction of the α-actin gene expression was found both in vivo and in vitro, suggesting the beginning of transdifferentiation process. Importantly, the in vitro findings suggest that the decrease of miR-145 probably preceded the α-actin reduction.

Recently, great interest has emerged in the study of miRs as regulators of several biological processes. Previous studies have shown the involvement of miRs in VSMCs transdifferentiation and vascular calcification processes [14,15,16]. Among them, miR-145 has been identified as the most abundant miR in normal arteries and isolated VSMCs, and it has been described that its expression is reduced in transdifferentiated VSMCs and injured arteries [20,38]. In addition, miR-145 has been able to drive the fate of pluripotent neural crest stem cells to VSMCs through the stimulation of the expression of numerous VSMCs differentiation markers, including α-actin, the marker used in the present study [19]. Previous studies in rats with CKD [21,39], showed severe arterial calcification and a decrease in the aortic and serum levels of miR-145, which paralleled the transdifferentiation of VSMCs and the decrease in α-actin expression. In addition, miR-145 inversely correlated with the Ca content and the severity of the vascular calcification in human arteries, positioning miR-145 as a good marker of vascular calcification [21]. 

The findings of the present study went a step forward and showed that the aortic and serum levels of miR-145 were the first phenotypic changes observed in the process of osteogenic transdifferentiation. This result has potential practical implications, because serum miR-145, which is easy to measure, showed a good correlation (r = 0.55, *p* = 0.012) with aortic miR-145 levels, giving the hope that in the future, measuring serum miR-145 levels may help to detect the initial steps of this complex process of vascular calcification. 

It has been suggested that high P could decrease miR-145 expression in human VSMCs and in aortas from ApoE deficient mice [16,21,40,41]. It is described that miR-145 regulates α-actin targeting different genes and acting by diverse mechanisms in the different cell types. It is described that miR-145 targets Gut-enriched Krüppel-like factor (GKLF, also called KLF4) and KLF5 that repress α-actin [20,42]. Other studies showed that miR-145 interacts with different Serum Response Factor (SRF), creating complex feedback loops to modulate actin and other VSMCs contractile markers [43]. The in vitro experiments performed in our study confirmed that P decreased α-actin and miR-145 expression and increased VSMCs calcification in a concentration-dependent manner. The decrease in miR-145 expression seems to be an early phenomenon; in fact, after 6 h of exposure of VSMCs to high P, the reduction in miR-145 expression was the first change observed, followed by the decrease in the expression of α-actin and later by the increase in Ca content. Several studies suggest that miRs are secreted from the cells in a free form or into vesicles as exosomes, suggesting a new form of intercellular communication that could regulate the phenotype of remote cells or tissues [44]. miR-145 levels increased in the culture medium probably due to the release by VSMCs, and its accumulation its lower with high P treatment.

To identify the role of any molecule in biological systems, its silencing and overexpression can provide relevant information. In fact, it has been shown that miR-145 silencing induced osteogenic transdifferentiation reducing the expression of the contractile markers α-actin, calponin and smooth muscle myosin heavy chain in VSMCs, while its overexpression increased their expression [17,20]. Similarly, in the present study, miR-145 silencing decreased α-actin expression (regardless the amount of P). On the other hand, miR-145 overexpression stimulated α-actin expression (also regardless of the amount of P), associated with the reduction in the calcification induced by high P. These data strongly suggest that miR-145 is essential to maintain the vascular phenotype in VSMCs and might protect them against calcification.

## 5. Conclusions

In summary, our in vivo and in vitro studies showed that high P induced sequential changes in the VSMCs phenotype with an early decrease in the levels of miR-145 that triggers the decrease in α-actin expression, inducing the loss of the contractile phenotype. Fortunately, serum levels and aortic expression of miR-145 are strongly correlated, and even though more studies are needed, our findings suggest that miR-145 may be an early biomarker of vascular calcification, able to detect the early steps of the transdifferentiation process of the VSMCs.

## Figures and Tables

**Table 1:** Serum and urinary biochemical parameters in rats fed a normal-P diet, (NP, 0.6% P) and high-P diet (HP, 0.9% P). *n*, number of rats. Fractional excretion was calculated as [(urine molecule × serum creatinine)/(serum molecule × urine creatinine)] × 100. Data represent median [interquartile range]. Bold data represents significant differences.

	NP	HP	*p*
*n*	10	10	
Serum P (mg/dL) (median [IQR])	3.98 [3.58, 4.18]	4.36 [4.12, 4.59]	**0.023**
Serum Ca (mg/dL) (median [IQR])	10.27 [10.06, 10.34]	10.17 [9.99, 10.31]	0.544
PTH (pg/mL) (median [IQR])	232.62 [171.18, 289.46]	459.62 [390.62, 618.39]	**<0.001**
FGF23 (pg/mL) (median [IQR])	55.55 [44.02, 124.57]	67.68 [40.96, 77.80]	0.796
Creatinine clearance (mL/min) (median [IQR])	3.00 [2.81, 3.25]	2.72 [2.20, 3.16]	0.151
Fractional Excretion of P (%) (median [IQR])	0.21 [0.09, 1.05]	21.77 [19.43, 24.77]	**<0.001**
Fractional Excretion of Ca (%) (median [IQR])	0.69 [0.54, 0.87]	0.25 [0.15, 0.30]	**0.003**

**Table 2:** Cardiovascular parameters (blood pressure, heart hypertrophy and aortic calcification) in rats fed a normal-P diet, (NP, 0.6% P) and high-P diet (HP, 0.9% P). *n*, number of rats. R.U. Relative Units. Data represent median [interquartile range]. Bold data represents significant differences.

	NP	HP	*p*
*n*	10	10	
Systolic Blood Pressure (mmHg) (median [IQR])	118.50 [116.34, 125.18]	125.39 [119.92, 129.14]	0.190
Diastolic Blood Pressure (mmHg) (median [IQR])	83.50 [78.63, 88.00]	93.86 [91.25, 98.54]	**0.015**
Heart weight (g)/Body weight (g) × 100 (median [IQR])	0.21 [0.20, 0.22]	0.21 [0.21, 0.21]	0.940
Aortic Calcium content (µg/mg protein) (median [IQR])	1.97 [1.39, 3.31]	4.88 [1.55, 6.49]	0.288
Aortas Von Kossa staining positive (%)	0	0	
Aortic α-actin expression (R.U.) (median [IQR])	1.03 [0.76, 1.90]	0.44 [0.37, 0.56]	**0.009**
Aortic *Runx2* expression (R.U.) (median [IQR])	0.79 [0.54, 1.34]	0.88 [0.66, 1.92]	0.485
Aortic *Osx* expression (R.U.) (median [IQR])	0.94 [0.40, 1.44]	1.71 [1.33, 1.94]	0.209

## Data Availability

The data underlying this article will be shared upon reasonable request to the corresponding author.

## Supplementary Materials

The following supporting information can be downloaded at: https://www.mdpi.com/article/10.3390/nu15132918/s1, Figure S1: Effect of different P concentrations (1–3.5 mM) on Runx2 (A) and Osterix (B) gene expression on A7r5 cells after 72 h of exposure. Figure S2: Effect of high P and different PTH concentrations (10^−9^–10^−7^ M) on miR-145 levels (A), α-actin gene expression (B) and Ca content (C) on A7r5 cells after 72 h of exposure in non-calcifying (1 mM P) and calcifying (3 mM P) medium.

## References

[1] Calvo M.S., Uribarri J. (2013). Public health impact of dietary phosphorus excess on bone and cardiovascular health in the general population. Am. J. Clin. Nutr..

[2] Hernández A., Concepción M.T., Rodríguez M., Salido E., Torres A. (1996). High phosphorus diet increases preproPTH mRNA independent of calcium and calcitriol in normal rats. Kidney Int..

[3] Zhou C., Shi Z., Ouyang N., Ruan X. (2021). Hyperphosphatemia and Cardiovascular Disease. Front. Cell Dev. Biol..

[4] Eddington H., Hoefield R., Sinha S., Chrysochou C., Lane B., Foley R.N., Hegarty J., New J., O’Donoghue D.J., Middleton R.J. (2010). Serum Phosphate and Mortality in Patients with Chronic Kidney Disease. Clin. J. Am. Soc. Nephrol..

[5] Vervloet M.G., Massy Z.A., Brandenburg V.M., Mazzaferro S., Cozzolino M., Ureña-Torres P., Bover J., Goldsmith D. (2014). Bone: A new endocrine organ at the heart of chronic kidney disease and mineral and bone disorders. Lancet Diabetes Endocrinol..

[6] Cannata-Andía J.B., Roman-Garcia P., Hruska K. (2011). The connections between vascular calcification and bone health. Nephrol. Dial. Transplant..

[7] Yamamoto K.T., Robinson-Cohen C., de Oliveira M.C., Kostina A., Nettleton J.A., Ix J.H., Nguyen H., Eng J., Lima J.A., Siscovick D.S. (2013). Dietary phosphorus is associated with greater left ventricular mass. Kidney Int..

[8] Chang A.R., Lazo M., Appel L.J., Gutiérrez O.M., Grams M.E. (2014). High dietary phosphorus intake is associated with all-cause mortality: Results from NHANES III. Am. J. Clin. Nutr..

[9] Sheridan K., Logomarsino J.V. (2017). Effects of serum phosphorus on vascular calcification in a healthy, adult population: A systematic review. J. Vasc. Nurs. Off. Publ. Soc. Peripher. Vasc. Nurs..

[10] Shroff R.C., Shanahan C.M. (2007). Vascular calcification in patients with kidney disease: The Vascular Biology of Calcification. Seminars in Dialysis.

[11] Mizobuchi M., Towler D., Slatopolsky E. (2009). Vascular Calcification: The killer of patients with chronic kidney disease. J. Am. Soc. Nephrol..

[12] Giachelli C.M. (2004). Vascular calcification mechanisms. J. Am. Soc. Nephrol..

[13] Moazed D. (2009). Small RNAs in transcriptional gene silencing and genome defence. Nature.

[14] Panizo S., Naves-Díaz M., Carrillo-López N., Martínez-Arias L., Fernández-Martín J.L., Ruiz-Torres M.P., Cannata-Andía J.B., Rodríguez I. (2016). MicroRNAs 29b, 133b, and 211 Regulate Vascular Smooth Muscle Calcification Mediated by High Phosphorus. J. Am. Soc. Nephrol..

[15] Goettsch C., Rauner M., Pacyna N., Hempel U., Bornstein S.R., Hofbauer L.C. (2011). miR-125b Regulates Calcification of Vascular Smooth Muscle Cells. Am. J. Pathol..

[16] Goettsch C., Hutcheson J.D., Aikawa E. (2013). MicroRNA in Cardiovascular Calcification: Focus on targets and extracellular vesicle delivery mechanisms. Circ. Res..

[17] Zhang C. (2009). MicroRNA-145 in vascular smooth muscle cell biology: A new therapeutic target for vascular disease. Cell Cycle.

[18] Taïbi F., Meuth V.M.-L., M’Baya-Moutoula E., Djelouat M.S.E.I., Louvet L., Bugnicourt J.-M., Poirot S., Bengrine A., Chillon J.-M., Massy Z.A. (2014). Possible involvement of microRNAs in vascular damage in experimental chronic kidney disease. Biochim. et Biophys. Acta BBA Mol. Basis Dis..

[19] Cordes K.R., Sheehy N.T., White M.P., Berry E.C., Morton S.U., Muth A.N., Lee T.-H., Miano J.M., Ivey K.N., Srivastava D. (2009). miR-145 and miR-143 regulate smooth muscle cell fate and plasticity. Nature.

[20] Cheng Y., Liu X., Yang J., Lin Y., Xu D.Z., Lu Q., Deitch E.A., Huo Y., Delphin E.S., Zhang C. (2009). MicroRNA-145, a Novel Smooth Muscle Cell Phenotypic Marker and Modulator, Controls Vascular Neointimal Lesion Formation. Circ. Res..

[21] Fernández-Villabrille S., Martín-Carro B., Martín-Vírgala J., Alonso-Montes C., Palomo-Antequera C., García-Castro R., López-Ongil S., Dusso A.S., Fernández-Martín J.L., Naves-Díaz M. (2023). MicroRNA-145 and microRNA-486 are potential serum biomarkers for vascular calcification. Nephrol. Dial. Transpl..

[22] Livak K.J., Schmittgen T.D. (2001). Analysis of relative gene expression data using real-time quantitative PCR and the 2^−ΔΔCT^ Method. Methods.

[23] Calvo M.S. (2000). Dietary considerations to prevent loss of bone and renal function. Nutrition.

[24] Calvo M.S., Moshfegh A.J., Tucker K.L. (2014). Assessing the Health Impact of Phosphorus in the Food Supply: Issues and Considerations. Adv. Nutr. Int. Rev. J..

[25] Kalantar-Zadeh K., Gutekunst L., Mehrotra R., Kovesdy C.P., Bross R., Shinaberger C.S., Noori N., Hirschberg R., Benner D., Nissenson A.R. (2010). Understanding Sources of Dietary Phosphorus in the Treatment of Patients with Chronic Kidney Disease. Clin. J. Am. Soc. Nephrol..

[26] Shinaberger C.S., Greenland S., Kopple J.D., Van Wyck D., Mehrotra R., Kovesdy C.P., Kalantar-Zadeh K. (2008). Is controlling phosphorus by decreasing dietary protein intake beneficial or harmful in persons with chronic kidney disease?. Am. J. Clin. Nutr..

[27] Sawin D.-A., Ma L., Stennett A., Ofsthun N., Himmele R., Kossmann R.J., Maddux F.W. (2020). Phosphates in medications: Impact on dialysis patients. Clin. Nephrol..

[28] Kendrick J., Kestenbaum B., Chonchol M. (2011). Phosphate and Cardiovascular Disease. Adv. Chronic Kidney Dis..

[29] Foley R.N., Collins A.J., Herzog C.A., Ishani A., Kalra P.A. (2009). Serum Phosphorus Levels Associate with Coronary Atherosclerosis in Young Adults. J. Am. Soc. Nephrol..

[30] Bai W., Li J., Liu J. (2016). Serum phosphorus, cardiovascular and all-cause mortality in the general population: A meta-analysis. Clin. Chim. Acta.

[31] Bozic M., Panizo S., Sevilla M.A., Riera M., Soler M.J., Pascual J., Lopez I., Freixenet M., Fernandez E., Valdivielso J.M. (2014). High phosphate diet increases arterial blood pressure via a parathyroid hormone mediated increase of renin. J. Hypertens..

[32] McClure S.T., Rebholz C.M., Mitchell D.C., Selvin E., Appel L.J. (2020). The association of dietary phosphorus with blood pressure: Results from a secondary analysis of the PREMIER trial. J. Hum. Hypertens..

[33] Palit S. (2014). Vascular Calcification in Chronic Kidney Disease: Role of Disordered Mineral Metabolism. Curr. Pharm. Des..

[34] Cozzolino M., Ciceri P., Galassi A., Mangano M., Carugo S., Capelli I., Cianciolo G. (2019). The Key Role of Phosphate on Vascular Calcification. Toxins.

[35] Carrillo-López N., Martínez-Arias L., Alonso-Montes C., Martín-Carro B., Martín-Vírgala J., Ruiz-Ortega M., Fernández-Martín J.L., Dusso A.S., Rodriguez-García M., Naves-Díaz M. (2021). The receptor activator of nuclear factor κΒ ligand receptor leucine-rich repeat-containing G-protein-coupled receptor 4 contributes to parathyroid hormone-induced vascular calcification. Nephrol. Dial. Transplant..

[36] Carrillo-López N., Panizo S., Alonso-Montes C., Martínez-Arias L., Avello N., Sosa P., Dusso A.S., Cannata-Andía J.B., Naves-Díaz M. (2019). High-serum phosphate and parathyroid hormone distinctly regulate bone loss and vascular calcification in experimental chronic kidney disease. Nephrol. Dial. Transplant..

[37] Owens G.K., Kumar M.S., Wamhoff B.R., Franco P.N., Durrant L.M., Carreon D., Haddad E., Vergara A., Cascavita C., Obenaus A. (2004). Molecular Regulation of Vascular Smooth Muscle Cell Differentiation in Development and Disease. Physiol. Rev..

[38] Ji R., Cheng Y., Yue J., Yang J., Liu X., Chen H., Dean D.B., Zhang C. (2007). MicroRNA Expression Signature and Antisense-Mediated Depletion Reveal an Essential Role of MicroRNA in Vascular Neointimal Lesion Formation. Circ. Res..

[39] Carrillo-López N., Panizo S., Arcidiacono M.V., de la Fuente S., Martínez-Arias L., Ottaviano E., Ulloa C., Ruiz-Torres M.P., Rodríguez I., Cannata-Andía J.B. (2022). Vitamin D Treatment Prevents Uremia-Induced Reductions in Aortic microRNA-145 Attenuating Osteogenic Differentiation despite Hyperphosphatemia. Nutrients.

[40] Rangrez A.Y., M’Baya-Moutoula E., Metzinger-Le Meuth V., Hénaut L., Djelouat M.S.E.I., Benchitrit J., Massy Z.A., Metzinger L. (2012). Inorganic Phosphate Accelerates the Migration of Vascular Smooth Muscle Cells: Evidence for the Involvement of miR-223. PLoS ONE.

[41] Rangrez A.Y., Massy Z.A., Meuth V.M.-L., Metzinger L. (2011). miR-143 and miR-145: Molecular keys to switch the phe-notype of vascular smooth muscle cells. Circ. Cardiovasc. Genet..

[42] Hu B., Wu Z., Liu T., Ullenbruch M.R., Jin H., Phan S.H. (2007). Gut-Enriched Krüppel-Like Factor Interaction with Smad3 Inhibits Myofibroblast Differentiation. Am. J. Respir. Cell Mol. Biol..

[43] Xin M., Small E.M., Sutherland L.B., Qi X., McAnally J., Plato C.F., Richardson J.A., Bassel-Duby R., Olson E.N. (2009). MicroRNAs miR-143 and miR-145 modulate cytoskeletal dynamics and responsiveness of smooth muscle cells to injury. Genes Dev..

[44] Chen X., Liang H., Zhang J., Zen K., Zhang C.-Y. (2012). Secreted microRNAs: A new form of intercellular communication. Trends Cell Biol..

