# Supplementary material for: Phosphorus May Induce Phenotypic Transdifferentiation of Vascular Smooth Muscle Cells through the Reduction of microRNA-145
Source: Nutrients. 2023 Jun 27;15(13):2918. doi: 10.3390/nu15132918 (PMC10346205; doi:10.3390/nu15132918)
Supplement: Supplementary file 1 [file nutrients-15-02918-s001.zip › nutrients-2437581-supplementary.pdf]

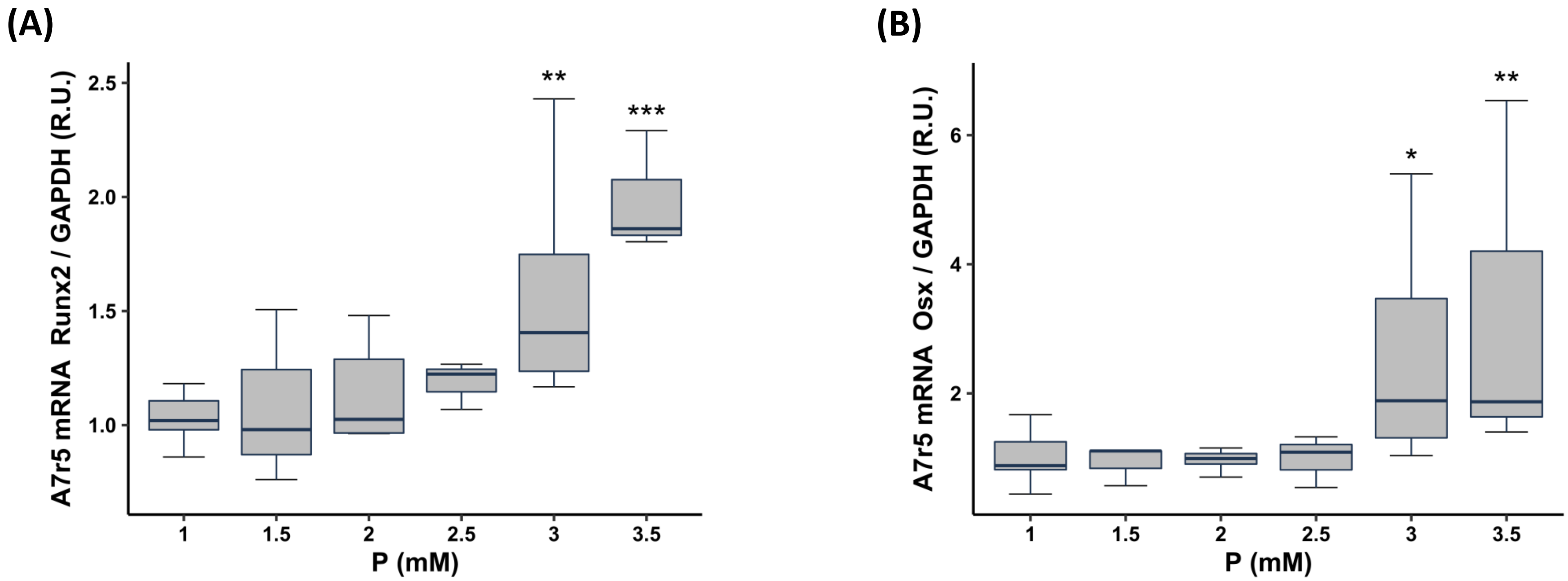

Supplementary Figure S1. Effect of different P concentrations (1-3.5 mM) on Runx2 (A) and Osterix (B) gene expression on A7r5 cells after 72 h of exposure. R.U. Relative units. Data represent median [interquartile range]. *p<0.05, **p<0.01, ***p<0.001 versus 1 mM P.

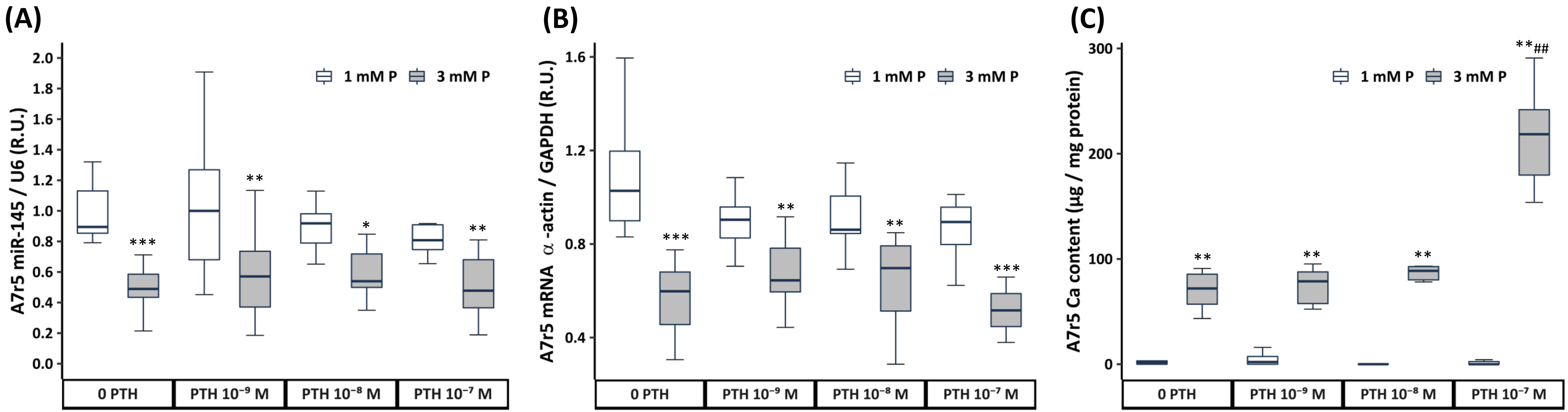

Supplementary Figure S2. Effect of high P and different PTH concentrations (10-9-10-7 M) on A7r5 cells after 72 h of exposure in non-calcifying (1 mM P) and calcifying (3 mM P) medium. miR-145 levels (A), α-actin gene expression (B) and Ca content (C). R.U. Relative units. Data represent median [interquartile range]. White and grey boxes represent non-calcifying and calcifying medium respectively. *p<0.05, **p<0.01, ***p<0.001 versus its own control (1 mM P). ##p<0.01 versus 1 mM P without PTH.
